# The role of ion channels and transporters in cell proliferation and cancer

**DOI:** 10.3389/fphys.2013.00312

**Published:** 2013-10-29

**Authors:** Andrea Becchetti, Luca Munaron, Annarosa Arcangeli

**Affiliations:** ^1^Department of Biotechnology and Biosciences, University of Milano-BicoccaMilano, Italy; ^2^Department of Life Sciences and Systems Biology, University of TorinoTurin, Italy; ^3^Department of Experimental and Clinical Medicine, University of FlorenceFlorence, Italy

**Keywords:** ion channels, cell cycle, proliferation, cancer, ion transporters

After the early wavering steps tracing back to at least the 1960's (Cone, 1974; Binggeli and Cameron, 1980), the study of ion transport in cell proliferation and neoplasia is on its way to become a mature research field (Arcangeli et al., 2009). Wide evidence is now available about the regulatory roles exerted by ion channels and transporters on the cell cycle phases (Becchetti, 2011) and other aspects of cell physiology that shape the multistep neoplastic progression, such as resistance to apoptosis (Lehen'kyi et al., 2011), cell invasiveness (Becchetti and Arcangeli, 2010), and angiogenesis (Fiorio Pla et al., 2012). Ion transport is implicated in these cell functions in many ways, from the classic mechanisms relating membrane potential (V_m_) to Ca^2+^ homeostasis, to the control of pH, cell volume, growth factor release, interaction with the extracellular matrix, and so forth. Some of these actions probably occur through non-conductive mechanisms, such as intrinsic enzyme activity or conformational coupling with other membrane proteins (e.g., Arcangeli and Becchetti, 2006; Hegle et al., 2006). A major function of ion channels is to mediate the cell interaction with its environment. In the case of cancer cells, the interaction with the tumor milieu' has relevant implications for therapy (Arcangeli, 2011).

The Research Topic Issue on *Ion Transport in Cell Cycle and Cancer* addresses classic issues in the field as well as it points to novel perspectives. One example of the latter is the role of Ca^2+^ in autophagy (Rizzuto et al., 2012), which has not received as much attention as Ca^2+^ signaling in the cell cycle machinery and apoptosis (Roderick and Cook, 2008; Munaron, 2012). As discussed by Kondratskyi et al. (2013), modulating autophagy has a great potential in cancer diagnosis and treatment. The review discusses the related Ca^2+^-dependent mechanisms and their meaning in the context of cancer progression and therapy. The role of Ca^2+^ in autophagy is related to the increasing recognition of the multiple functions of intracellular ion channels, which regulate ion transport across the membranes of cellular organelles. Leanza et al. (2013) provide an updated overview of the role of ion channels in mitochondria, endoplasmic reticulum, nucleus, endosomes, and lysosomes, with special attention to the implications for the biology of cancer.

A general picture of the role of V_m_ in cell proliferation and differentiation is provided by Yang and Brackenbury (2013), with a special focus on voltage-gated channels. The paper reviews what is known about the V_m_ changes during cell cycle and the implications of different types of ion channels in the cell cycle stages, cancer cell migration and the differentiation of cancer stem cells. That a wide variety of ion channel types affect the main hallmarks of cancer is also suggested by Crottès et al. (2013), who focus on the role of the sigma-1 receptor (Sig1R) in cancer cells. Sig1R is a stress-activated chaperone associated with both the plasma membrane and the interface between the mitochondria and the endoplasmic reticulum. Sig1R is often expressed in tumors and several lines of evidence suggest that it is implicated in regulating Ca^2+^ homeostasis as well as some of the major types of ion channels. The authors suggest that Sig1R contributes to regulate the ion channel expression and function in cancer cells in response to environmental signals.

Following the lead of the seminal studies in lymphocytes (DeCoursey et al., 1984) and early embryos (Day et al., 1993), K^+^ channels continue to have the lion's share in the field. An overview of the general role exerted by K^+^ channels in cell cycle progression is provided by Ouadid-Ahidouch and Ahidouch (2013), whereas the specific contribution of the voltage-gated K_V_1.3 and K_V_1.5 channels in human cancer is reviewed by Comes et al. (2013). A group of voltage-gated K^+^ channels frequently found to be involved in cancerogenesis comprises the *ether-à-go-go* (K_V_10 or Eag; Pardo and Stühmer, 2008) and *ether-à-go-go-related* (K_V_11 or Erg; Arcangeli, 2005) subtypes, whose cell biology is under intense investigation. In this issue, Herrmann et al. (2013) show that the surface expression of the oncogenic K_V_10.1 channel is regulated by the Golgi-resident protein PIST (also known as GOPC), by an interaction mediated by PDZ domain.

In addition, growing evidence points to the role of ligand-gated channels in cancer cells. In particular, the nicotinic acetylcholine receptors (nAChRs) are homo- or heteropentamers of α and β subunits. These were originally identified in the nervous system, but are now increasingly recognized to be widely expressed outside the nervous system (Egleton et al., 2008; Schuller, 2009; Ambrosi and Becchetti, 2013). Thus, Improgo et al. (2013) discuss the role of the nAChR genes in lung cancer and propose an interesting mechanism whereby signaling mediated by α3/α5/β4-containing nAChRs (which genome-wide analyses correlate with increased smoking dependence and risk of developing lung tumors) promote carcinogenesis in small cell lung carcinoma cells.

Another central aspect of the function of normal and neoplastic cells is the regulation of cell volume, as reviewed by Pedersen et al. (2013). This applies especially to the secretive and absorbing epithelia, in which the massive transport of ions, organic compounds, and fluid must be tightly matched to the control of cell volume. Such regulatory processes are generally disrupted in cancer cells and the general oncologic relevance of these observations turns on the fact that most cancers derive from epithelial cells. The authors give a thorough overview of the interaction between the tumor microenvironment and the altered regulation of ion transport. Moreover, they discuss the role of cell volume in cell proliferation and apoptosis, and the involvement of ion transport in tumor drug resistance, with special focus on the implication of chloride, calcium, and pH regulation.

A key step of the neoplastic progression is the regulation of cell migration, whose derangement is implicated in the metastatic cascade (Becchetti and Arcangeli, 2010) and tumor vascularization (Fiorio Pla et al., 2012). Some subtypes of the Transient Receptor Potential (TRP) channels have been found to participate to the regulation of cell migration, as is reviewed by Fiorio Pla and Gkika (2013). The authors especially focus on the implication of TRP channels in cell migration during the neoplastic progression. A different and novel aspect of the regulation of cancer cell migration is the implication of the tumor-associated carbonic anhydrase IX (CA IX) on focal contacts during cell spreading and migration. CA IX provides intracellular bicarbonate and extracellular H^+^ to support cancer cell survival and invasiveness and is the only human CA isoform containing an extracellular proteoglycan domain (Monti et al., 2012). In their paper, Csaderova et al. (2013) show the regulatory interaction of CA IX with the focal contact sites, and provide the first evidence that CA IX localizes in the focal adhesion structures.

Several of the above papers describe the potential therapeutic applications of targeting specific ion channels and transporters. Cancer treatment is however more specifically addressed by Huber et al. (2013), who illustrate the use of ionizing radiation. This causes double-strand DNA breakages and thus cancer cell death, but also targets the plasma membrane. The ensuing modifications of ion channels and transporters can contribute to the survival of the irradiated cells. The authors discuss what is known about the mechanisms of the radioresistance dependent on ion transporters and suggest possible ways to make tumor cells more sensitive to radiation by proper targeting of ion channels and transporters.

Besides illustrating some of the hot topics in the field, the papers of the present Research Topic Issue constitute a most useful introduction to a literature that has already become too vast to be mastered by a single investigator.
